# Biomethane Production From Lignocellulose: Biomass Recalcitrance and Its Impacts on Anaerobic Digestion

**DOI:** 10.3389/fbioe.2019.00191

**Published:** 2019-08-08

**Authors:** Ning Xu, Shixun Liu, Fengxue Xin, Jie Zhou, Honghua Jia, Jiming Xu, Min Jiang, Weiliang Dong

**Affiliations:** ^1^State Key Laboratory of Materials-Oriented Chemical Engineering, College of Biotechnology and Pharmaceutical Engineering, Nanjing Tech University, Nanjing, China; ^2^Jiangsu Key Laboratory for Biomass-Based Energy and Enzyme Technology, Huaiyin Normal University, Huai'an, China; ^3^Jiangsu National Synergetic Innovation Center for Advanced Materials (SICAM), Nanjing Tech University, Nanjing, China

**Keywords:** biomethane, anaerobic digestion, lignocellulose, cell wall composition, biomass recalcitrance

## Abstract

Anaerobic digestion using lignocellulosic material as the substrate is a cost-effective strategy for biomethane production, which provides great potential to convert biomass into renewable energy. However, the recalcitrance of native lignocellulosic biomass makes it resistant to microbial hydrolysis, which reduces the bioconversion efficiency of organic matter into biogas. Therefore, it is necessary to critically investigate the correlation between lignocellulose characteristics and bioconversion efficiency. Accordingly, this review comprehensively summarizes the anaerobic digestion process and rate-limiting step, structural and compositional properties of lignocellulosic biomass, recalcitrance and inhibitors of lignocellulose and their major effects on anaerobic digestion for biomethane production. Moreover, various type of pretreatment strategies applied to lignocellulosic biomass was discussed in detail, which would contribution to cell wall degradation and improvement of biomethane yields. In the view of current knowledge, high energy input and cost requirements are the main limitations of these pretreatment methods. In addition to optimization of fermentation process, further studies should focus much more on key structural influence factors of biomass recalcitrance and anaerobic digestion efficiency, which will contribute to improvement of biomethane production from lignocellulose.

## Introduction

Lignocellulose is one of the most abundant renewable organic resources with an increasing annual yield of 200 billion tons, which can be produced from agriculture, forestryand urban wastes (Patinvoh et al., 2017). The prominent abundance and low cost of lignocellulose make it a potential substrate for second generation bioenergy production, such as bioethanol and biomethane (Florian et al., 2013). During these, biomethane production is one of the most cost-effective methods for energy generation from lignocellulosic cellulose, which has been implemented worldwide (Grosser, 2017).

**Figure 1:**
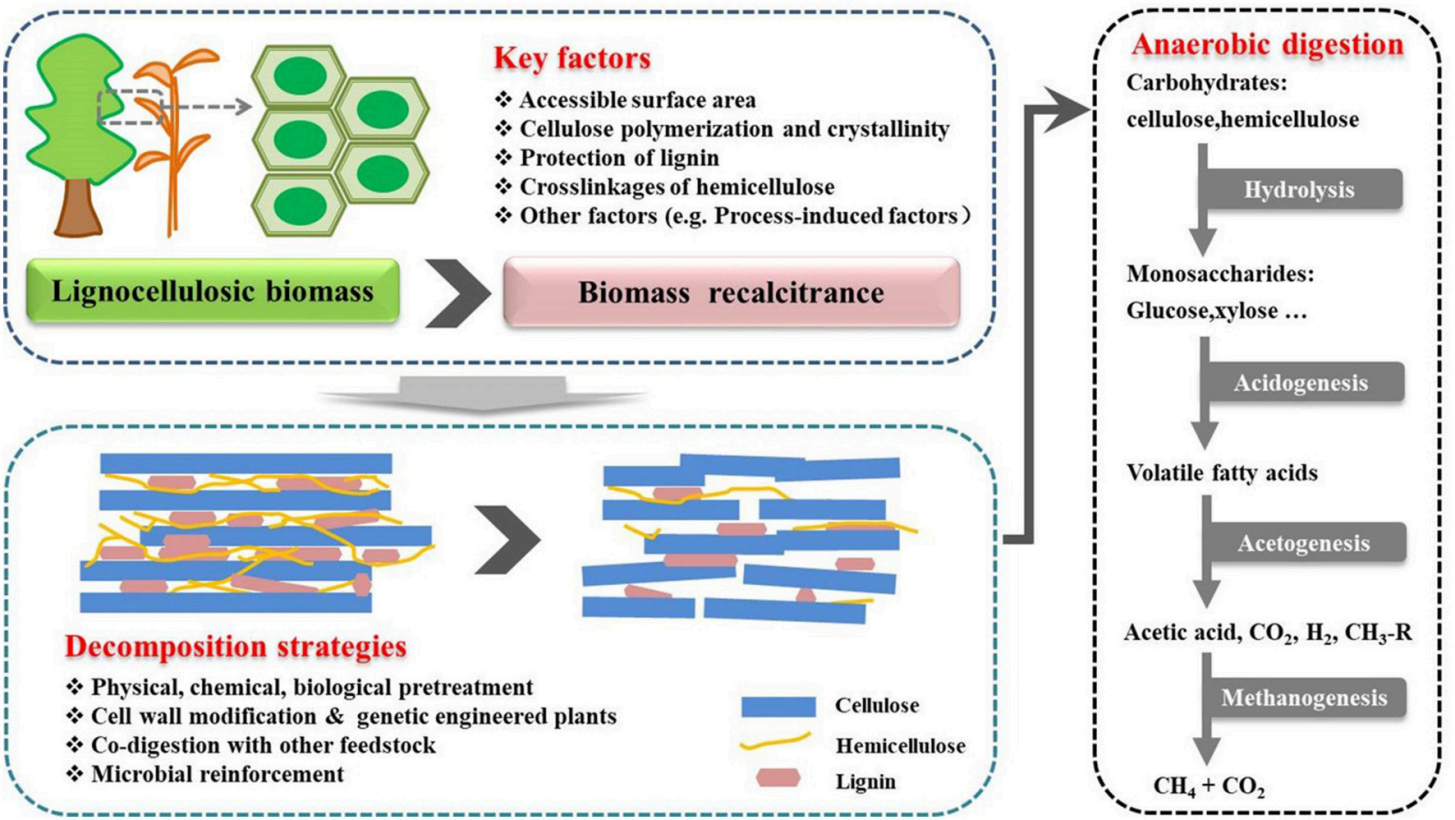
Process stages of the conversion of lignocellulosic biomass to biomethane. Biomethane production is a naturally occurring biological process, which can be divided into four stages. Recalcitrance of lignocellulose restricts the hydrolysis during the first stage. Pretreatment is necessary step for biomethane production. The positive effects of pretreatment strategies can help to facilitate the hydrolysis of lignocellulosic in the first stage (Florian et al., 2013; Hagos et al., 2016).

Biomethane production through anaerobic digestion is a naturally occurring biological process, which can be divided into four steps (Figure 1). In the beginning of the process, complex organic polymers are decomposed to their component units, e.g., amino acids, fatty acids, and sugars, respectively. Then, these monomers are converted into a mixture of short chain volatile fatty acids by fermentative bacteria (Acidogens). Acetogenic bacteria or acetogens further convert the volatile fatty acids to acetate, carbon dioxide, and hydrogen, which are natural substrates for Methanogenesis to generate biomethane. Theoretically, AD process can decompose the organic fraction of any feedstocks to produce biomethane, such as crop and livestock residues, food waste and lignocellulosic feedstocks (Hagos et al., 2016). However, methane production varies greatly with different types of substrates. For example, high methane yields up to 450 mL CH_4_/g volatile solids can be achieved with sugar and starch crops (Frigon and Guiot, 2010), while no more than 330 mL CH_4_/g volatile solids can be produced from lignocellulosic biomass (Table 1). The complexity of biomass structure is the major challenge, which makes lignocellulosic biomass highly recalcitrant to anaerobic degradation and ultimately results in low biomethane yield (Sawatdeenarunat et al., 2016). The stubborn anti-degradation characteristics of native lignocellulose was known as biomass recalcitrance, which extremely restricts the hydrolysis during the first step of anaerobic digestion process and finally limits the commercial biomethane production from lignocellulose (Himmel et al., 2007).

**Table 1:** Biomethane production of selected lignocellulosic biomass.

**Biomass**	**Inoculum**	**Operation conditions**	**Methane production**	**References**
Hydrolysis lignin (lignin content of 80%) of Birch wood chips	CSTR^c^ running with food waste and cow manure	37°C, 90 rpm, 39 days	125 mL CH_4_/g VS	Mulat et al., 2018
Paper paste	Anaerobic sludge	Pretreated with cellulolytic microbial consortium, then pH 7.3, 55°C, 90 days	101 mL CH_4_/g cellulose	Kinet et al., 2015
Rice straw	Anaerobic sludge	Fungal pretreatment, then SS-AD reactors, 37 ± 1°C for 45 days	152~263 mL CH_4_/g VS	Mustafa et al., 2016
Reed canary grass (Cultivated and wild)	Sewage sludge	35 ± 1°C, pH 7.0, 20~40 days	Cultivated: 406 ± 21; Wild: 120 ± 16 mL CH_4_/g VS	Oleszek et al., 2014
Miscanthus. *giganteus*	Mesophilic digestate	35°C, 90 days	285~333 mL CH_4_/g VS	Wahid et al., 2015
Miscanthus. *sinensis*	Mesophilic digestate	35°C, 90 days	291~320 mL CH_4_/g VS	Wahid et al., 2015
Switchgrass (WHS^a^)	–	Different pretreatment (G^d^, GA^e^, GAA^f^), 35°C anaerobic fermentation for 38 days	G: 112.4 ± 8.4; GA: 132.5 ± 9.7; MA A:139.8 mL CH_4_/g VS	Frigon et al., 2011
Switchgrass (SHS^b^)	–	Different pretreatment (C^g^, M^h^, MA^i^), 35°C anaerobic fermentation for 36 days	C: 94.7 ± 4.4; M: 152.3 ± 1.2; MA: 256.6 ± 8.2 mL CH_4_/g VS	Frigon et al., 2011
Barley	AD reactor digesting cattle slurry and grass silage	37°C, 35 days	314.8 mL CH_4_/g VS	Himanshu et al., 2018
Wheat straw	Anaerobic sludge	Laccase, versatile peroxidase pretreatment, then 37°C anaerobic fermentation for 30 days	250.5 mL CH_4_/g VS	Schroyen et al., 2015
Sunflower	Digestate	35°C, pH 8.1 ± 0.3, 30 days	210~286.1 mL CH_4_/g ODM^j^	Herrmann et al., 2016
Sorghum	Digestate	35°C, pH 8.1 ± 0.3, 30 days	298.9~311.3 mL CH_4_/ g ODM^j^	Herrmann et al., 2016
Corn straw	Biogas slurry	55°C and 5 ml/g O_2_ pretreatment, then 37°C anaerobic fermentation	325.7 mL CH_4_/g VS	Fu et al., 2015

a *WHS, winter harvested Switchgrass*.

b *SHS, summer harvested Switchgrass*.

c *CSTR, continuously stirred tank reactor*.

d *G, ground*.

e *GA, ground and alkalinization*.

f *GAA, ground, alkalinization and autoclaving*.

g *C, chopped*.

h *M, mulched*.

i *MA, mulched and alkalinization*.

j *ODM, organic dry matter*.

In order to overcome this recalcitrance, lignocellulose must be pretreated and many pretreatment methods have been developed in recent years. The positive effects of various pretreatments (e.g., increase of surface area, lignin removal, decrease of cellulose crystallinity) have been reviewed elsewhere (Paudel et al., 2017). However, an overall review and assessment about the impacts of lignocellulose recalcitrance on anaerobic digestion and biomethane production is still needed and imperative for further biomethane development. Hence, the aim of this paper is to provide a comprehensive review of lignocellulose recalcitrance and its relative effects on anaerobic fermentation and biomethane production. In addition, the technology for acceleration of anaerobic digestion of lignocellulose and future prospective was also discussed.

## Basic Structural Properties of Plant Cell Wall and Lignocellulose Recalcitrance

Lignocellulosic biomass is mainly composed of cellulose, hemicellulose and lignin, which vary a lot based on types of plants, growth conditions and maturation both in quantity and quality (Table 2). The detailed structure has been comprehensively reviewed elsewhere (Jeoh et al., 2017). Biomass recalcitrance refers to the anti-degradation characteristics of native lignocellulose, which protect plant cell wall from pathogen attack or degradation by microorganisms and enzymes. It was caused by the complicated compositions and structure of plant cell wall (Figure 1). Cellulose is a relative homogeneous substance in terms of the composition and structure, which provides the basic backbone to lignin-carbohydrate complexes. Hemicelluloses are embedded through the cell wall and form covalent bonds to the surface of cellulose fibrils (Somerville et al., 2004), which help strengthen the cell wall. As a filler compound, lignin wrappers itself in the interspace of cellulose and hemicellulose chains and formed a hydrophobic lignification structure, which plays an important role in maintaining the structural integrity of the cell wall (Yuan et al., 2013). Besides the three main compositions, cell wall proteins, lipids, pectin, mineral and other matters are also involved in the formation of biomass recalcitrance. Moreover, in addition to chemical composition and physical structure, the arrangement and density of the vascular bundles, epidermal protection and some process-induced causes also play considerable role in building the cell wall matrix.

**Table 2:** Biochemical composition of selected lignocellulosic biomass (w/w, %).

**Biomass**	**Cellulose**	**Hemicellulose**	**Lignin**	**References**
Sunflower stalk	31.0	15.6	29.2	Monlau et al., 2012
Barley straw	34.3	23.0	13.3	Saha and Cotta, 2010
Wheat straw	35.0	22.3	15.6	Boladorodríguez et al., 2016
Miscanthus	38.2	24.3	25.1	Vrije et al., 2002
Rice straw	38.6	19.7	13.6	Zhu et al., 2005
Pine	43.3	21.5	28.3	Florian et al., 2013
Polar	44.5	22.5	19.5	Florian et al., 2013
Corn straw	45.4	22.7	10.8	Fu et al., 2015
Spruce	45.5	22.9	27.9	Florian et al., 2013
Eucalyptus	54.1	18.4	21.5	Florian et al., 2013

As discussed above, biomass recalcitrance refers to lignocellulosic building blocks which are naturally evolved to block their microbial and enzymatic deconstruction. This is the result of a sophisticated combination of the crystalline cellulose in microfibrils, heteropolysaccharides, lignin, and other components (Table 3). In the first step of anaerobic digestion process (Figure 1), biomass recalcitrance protects itself from degradation by microorganisms and enzymes, which result in lower monosaccharide production and finally limits the biomethane efficiency. It is known that the degree of recalcitrance varies depending on the composition of the lignocellulosic biomass, which closely correlated to genotype, environmental conditions, crop management practices and plant parts (Surendra et al., 2018). However, there are some basic components and major influencing factors which generally exist in different plants. The detailed of these properties and its impacts on anaerobic digestion for biomethane production are discussed in the following.

**Table 3:** Different factors constructing biomass recalcitrance.

**Factors**	**Relative effects**	**References**
Epidermal protection	The epidermal tissue of the plant body, particularly the bark, cuticle and epicuticular waxes	Greenshields et al., 2004; Zhao et al., 2012
Cellulose characteristic	High degree of CrI and DP of cellulose, challenges for enzymes acting on insoluble substrate	Himmel et al., 2007; Zhang et al., 2013
Chemical compositions	Heterogeneity and complexity of constituents, degree of lignification, and complexity of chemical cross-linkages	Karimi and Taherzadeh, 2016
Cell wall physical structure	Arrangement and density of the vascular bundles; the relative amount of sclerenchymatous tissue	Vogel, 2008; Zhang et al., 2013
Process-induced causes	Inhibitors are generated during conversion processes (e.g., cellulose realignment)	Himmel et al., 2007

### Accessible Surface Area of Cellulose

Accessible surface area of substrate refers to the surface area, by which cellulases can contact with cellulose. In anaerobic digestion process, it could directly affect the biodegradability of lignocellulosic materials, which limits the contact between lignocellulose and enzyme, microbial or chemical reagents and result in insufficient fermentable sugars for the subsequent process (Kratky and Jirout, 2015). Accessible surface area can be affected by many indirect factors, e.g., epidermal feature, particle size of raw material powder, chemical and physical characteristics of plant cell wall (Florian et al., 2013). Accessible surface area can be divided into two forms: interior surface area which is determined by substrate porosity and exterior surface area which is correlated with particle size (Zhao et al., 2012). Generally, natural lignocellulosic substrates have very small interior surfaces, especially for dried material (Park et al., 2006). Arantes and Saddler (2011) have reported that cellulose accessibility to enzymes or chemical regents is mainly through the inside pores of substrate (about 90%) rather than the external surface, suggesting that the external surface only plays less important role in hydrolysis progress.

Lignocellulosic biomass is hydrolyzed by hydrolytic bacteria to release saccharides for biomethane production. These microorganisms will bind to the lignocellulose surface through physical contact, and then secrete extracellular multi-enzyme complexes to initiate the hydrolysis. Accessible surface area is considered as an important factor for the biodegradability of lignocellulosic materials and the substrate should have enough pores for efficient hydrolysis (Karimi and Taherzadeh, 2016). Generally, the diameter of the pore ranged from 0.2 to 20 μm, which is similar to the size of the bacteria. During the anaerobic digestion progress, the accessible surface area will increase along with the removal of partial cell wall component, resulting in higher surface availability. However, enzymatic hydrolysis is usually faster at the beginning and slower in the latter stages (Vivekanand et al., 2014), indicating that the surface area is not the only controlling factor for the hydrolysis. At the initial stage, lager surface area allows sufficient contact between enzymes and digestible amorphous cellulose, resulting in faster hydrolysis. But in the later period of anaerobic digestion, even though the accessible surface area is increasing, the remaining higher crystalline cellulose and the compact structure become the main factors which finally limit the hydrolysis efficiency (Khodaverdi et al., 2012).

### Cellulose Polymerization and Crystallinity

Degree of cellulose polymerization referring to the molecular weight of cellulose chains is an important factor affecting the enzymatic hydrolysis of cellulose. In the last few decades, many methods have been developed to give more accurate polymerization degree of cellulose (Hubbell and Ragauskas, 2010). It is known that the enzymatic hydrolysis of cellulose is the depolymerization process of cellulose by cellulase, which is directly related with cellulose polymerization degree. With the prominent reduction of cellulose polymerization degree from 247 to 151, steam explosion pretreatment yields 5–6 folds enhancements of enzymatic saccharification (Huang et al., 2015). Generally, more intramolecular hydrogen bond in long cellulose chains will hinder the cellulose conversion compared to shorter ones (Karimi and Taherzadeh, 2016). According to Waliszewska et al. (2018), the partial cellulose with lower polymerization was hydrolyzed preferentially in anaerobic digestion; resulting in the increase of cellulose polymerization degree after the methane fermentation process.

Cellulose crystallinity refers to the proportion of crystalline region of cellulose, which generally ranges from 30 to 80%. Hydrogen bonds and van der Waals forces are main acting forces to form crystalline structure (Zhang et al., 2013). Cellulose chains have different orientations, leading to three different levels of crystallinity including crystalline, sub-crystalline and amorphous forms. There are several crystalline and non-crystalline regions in microfibrils, however, no obvious boundary exists between different regions (Park et al., 2010). The crystallization zone is characterized by the good chain orientation, compact arrangement, high density and strong intermolecular bonding. The non-crystalline region is characterized by the poor chains orientation of cellulose, unordered molecular arrangement, large distance between molecules, low density and less hydrogen bonding between molecules (Park et al., 2010). Because of the high endo-glucanase activity of cellulase with the amorphous (non-crystalline) region, cellulose crystallinity plays noticeable role in affecting initial hydrolysis of cellulose. The yield of monosaccharides decreased with the increased crystallinity of the substrate, indicating that amorphous domains are hydrolyzed first before the hydrolysis of crystalline parts (Zhe et al., 2017). Mirahmadi et al. (2010) found that alkaline pretreatment with NaOH resulted in the significant reduction of crystallinity, which improved enzymatic hydrolysis and led to 83 and 74% improvement in methane production from birch and spruce.

In order to understand the mechanism of impacts of cellulose crystallinity on cellulose hydrolysis, several functional quantitative models have been designed. Jeoh et al. (2017) pointed out that cellulose crystallinity greatly impacted the adsorption of cellobiohydrolase Cel7A (CBHI), which resulted in lower cellulase hydrolysis efficiency. Moreover, with constant concentration of adsorbed enzyme, the initial enzymatic hydrolysis rate decreased with increasing cellulose crystallinity, which means that cellulose crystallinity can also affect the effectiveness of adsorbed cellulase components (Hall et al., 2010). In addition, different cellulase components showed different capacities and activities of adsorption with various cellulose forms (Zhang and Lynd, 2004). For example, endoglucanase I showed greater capacity of adsorption than CBHI and its higher crystallinity resulted in increasing adsorption of a non-hydrolytic protein named fibril-forming protein from *Trichoderma reesei* (Ding and Xu, 2004).

Cellulose is the most important component of plant cell wall, and the negative effect of cellulose polymerization degree and cellulose crystallinity on enzymatic hydrolysis has been recognized as mentioned above. However, more investigation is needed regarding the various cellulose properties and parameters, e.g., changes of cellulose structure during fermentation process (Waliszewska et al., 2018), the cellulase adsorption and desorption (Yang et al., 2011), combined effect of cellulose and other cell wall properties (Jeoh et al., 2017).

### Crosslinkages of Hemicellulose and Lignin

In contrast to cellulose, hemicellulose is a branched polysaccharide consisting of various sugar units. Xylan, the backbone chains of 1, 4-linked β-d-xylopyranose is the most abundant component of hemicellulose. The matrix properties of hemicellulose are complicated and significantly influenced by crosslinking agents (e.g., ferulic acid), monosaccharides characteristics and abundance of side chains (Somerville et al., 2004; Vogel, 2008).

It is generally believed that hemicellulose can increase the structural strength of plant cell wall and the space resistance, resulting in decreased hydrolysis efficiency. Pretreatment can effectively remove or dissolve lignin and hemicellulose, thereby increase the accessibility of the cellulose to microorganisms or enzymes (Hendriks and Zeeman, 2009). By carefully controlling the solids retention time, methane production can be enhanced from hemicellulose exclusively, while cellulose and lignin are left over in the residues. For anaerobic bioconversion of lignocellulose, hemicellulose was commonly removed earlier which decreased the structural obstacle degree for downstream enzymatic hydrolysis. Therefore, some result indicated that hemicelluloses were might be a positive factor to promote biomass digestibility by negatively affecting lignocellulosic recalcitrance. Our previous study suggested that the hemicellulose branch connected to the cellulose crystalline region and construct the non-crystalline region, thus positively reduce the crystallinity of cellulose, resulting in much more easy hydrolysis site and higher hydrolysis efficiency of cellulose consequently (Xu et al., 2012). Moreover, branched arabinose (Ara) might be an important influence factor, which could build interlinking (β-1, 4-glucans) to cellulose fibers to decrease cellulose crystallinity, and would improve the saccharification efficiency (Li et al., 2015). In a word, because of the complexity of hemicellulose structure and cross-linking between cell wall components, more research is still needed to carefully interpret the hemicellulose properties and its effect on methane production.

Lignin is a complex polymer of phenylpropane units that form a three-dimensional network inside the cell wall. It is generally considered to be the most important factor which limiting the biodegradability of lignocellulose. Studies have shown that 1% increase of lignin content would result in an average reduction of 7.49 L CH_4_/kg total solid (Thomsen et al., 2014). Moreover, Triolo et al. (2012) found that the excess of lignin (>100 g/kg volatile solid) would result in notable lower methane yield. Lignin restricts the degradation of structural polysaccharides by hydrolytic enzymes, thereby limiting the bioconversion of lignocellulose (Ahring et al., 2015). Generally, two main mechanisms have been proposed to illustrate this phenomenon. First, lignin consolidates the cell wall structure by covalent linkages with other cell wall components, which increases space resistance and prevents the carbohydrate from enzymatic hydrolysis (Yuan et al., 2013). A comparison between woody materials and grass revealed that the higher abundant of covalent linking and the phenyl groups in lignin result in harder digestion in wood than grass (Ververis et al., 2004). Moreover, the lignin structural units also have influence in biomass degradation efficiency. A previous study reported that different contents of three lignin monolignols (Syringyl, Guaiacyl, and *p*-Hydroxyphenyl), syringyl/guaiacyl ratio and interlinked-phenolics could affect enzymatic digestion after NaOH and H_2_SO_4_ pretreatments (Li et al., 2014).

Another influence of lignin is its adsorption capacity to enzymes (Lu et al., 2016). Lignin can affect enzymatic hydrolysis by non-specific or non-productive adsorption of cellulase (Palonen et al., 2004). The adsorption of cellulase to lignin has been mediated by three mechanism: hydrogen bonding (Berlin et al., 2006), hydrophobic (Eriksson et al., 2002) and electrostatic interactions (Nakagame et al., 2011). In lignocellulose digestion progress, three types of interactions may be involved in the non-productive adsorption of cellulases to lignin. Most studies suggested that hydrophobic interaction is the major cause for non-productive adsorption of enzyme to lignin, while less attention has been paid on hydrogen bonding and electrostatic interactions. However, until now, it is difficult to point out which one is the dominant in the specific reaction because of complex structure of different substrate (Saini et al., 2016). Electrostatic action was a main factor influencing the adsorption of endo-beta-1, 4-glucanases and xylanase onto lignin, while hydrophobicity mainly affected the adsorption of cellobiohydrolases and β-Glucosidase onto lignin (Lu et al., 2016). Thermodynamic analysis of enzyme adsorption onto lignin indicated that the adsorption was a spontaneous process and higher temperature would accelerate the process (Tu et al., 2009). This provides enlightenment that the enzymatic hydrolysis should be conducted at as low temperature as possible to avoid cellulase adsorption with lignin.

### Non-structural and Other Factors Restricting Lignocellulose Degradation

Besides physical and chemical characteristics of cell wall as mentioned above, there are also some other factors which may reduce lignocellulose biodegradation. For example, bioconversion processes may generate some additional inhibitors or negative variation of cell wall structure. Reduction of article size to 0.36–0.55 mm and 0.71–1.0 mm could achieve lower methane yield when compared with size of 1.4–2.0 mm (Rubia et al., 2011). This result might be attributed to inhibitors (e.g., overproduction of volatile fatty acid) and chemical transformation generated from excessive particle size reduction. Moreover, delignification beyond 50% might cause collapse of cellulose matrix, resulting in compact and chaotic structure and subsequent decrease in cellulose accessibility (Zhu et al., 2008). In addition, the crystal structure of cellulose can be transformed. For example, alkali extraction can transform cellulose I to cellulose II, and cellulose II are antiparallel configuration which generally do not exist in the natural cell wall (Zhang et al., 2013). Such structural changes or hazardous substances caused by the pretreatment processes are additional inhibitors to anaerobic digestion, and should be taken into consideration as part of the biomass recalcitrance. But compared with the native structures and characteristics of the plant cell wall, these additional inhibitors are by-products of the process of cracking cell wall recalcitrance and are just minor contributors to restrict the fermentation efficiency. In the process of biomethane production from lignocellulose, the ideal process strategy is efficiently breaking down the lignocellulosic recalcitrance while minimizing the production of by-products.

## Strategies to Overcome Recalcitrance for Higher Biomethane Production

Pretreatment prior to biomethane fermentation is an effective method to reduce the biomass recalcitrance and increase the accessibility in AD (Weiland, 2010). Recent years, many studies have provided various physical, chemical or biological pretreatments in the production of biomethane, and their major effects are summarized in Table 4.

**Table 4:** Conventional pretreatments and notable effects.

**Pretreatment**	**Notable effects**	**References**
Grining/milling	Size reduction, larger surface area and pore size, lower crystallinity	Kratky and Jirout, 2015
Irradiation	Cleavage of chemical bonds, lager surface area	Siddique et al., 2017
Steam explosion	Increase of surface area and pore size, solubilization of hemicellulose	Zhou et al., 2016
Liquid hot water	Lager surface area, solubilization of hemicellulose	Yu et al., 2013
Alkali	Cleavage of lignin, dissolution of hemicellulose, increase of internal surface area, reduction of polymerization	Boladorodríguez et al., 2016
Acid	Hydrolysis of hemicellulose, alteration of cellulose structure, larger surface area	Zhou et al., 2014
Oxidizing agents	Removal of hemicellulose and lignin, increase of cellulose accessibility	Florian et al., 2013
Organic solvent	Solubilization of hemicellulose or lignin, larger surface area	Zheng et al., 2014
Ammonia fiber explosion	Solubilization of lignin, disruption of LCC structure, increase of cellulose accessibility	Huang et al., 2015; Zhou et al., 2016
Ionic liquids	Solubilization of cellulose, reduction of crystallinity	Xu et al., 2016; Cao et al., 2017
Fungal	Delignification and partial hydrolysis of hemicellulose, alteration of LCC structure	Kudanga and Roes-Hill, 2014

### Physical Pretreatment of Lignocellulose

Physical (mechanical) pretreatment refers to the pretreatment processes without chemicals or microorganisms, which includes comminution (e.g., milling and grinding), irradiation (e.g., ultrasound, gamma ray, and microwave), steam explosion, liquid hot water pretreatment and others.

Comminution is mainly used to reduce the particle size, which increases the accessible surface area, alters the ultrastructure, and reduces the cellulose crystallinity and polymerization degree of cellulose for improved digestibility (Kratky and Jirout, 2015). Generally, comminution is the most common pretreatment method and always the first step ahead of the whole biomethane production process. Biogas production would be increased with the reduction of particle size. However, to the different lignocellulose compositions of the various particle size ranges, excessive particle size reduction may produce inhibitors and decrease biogas production (Rubia et al., 2011). Therefore, particle size should be carefully considered when different lignocellulose substrate was employed. Irradiation could preferentially dissociate the glucoside bonds of the cellulose and degrade cellulose chains into brittle fibers, oligosaccharides, or even cellobiose. Siddique et al. (2017) found that microwave and ultrasonic pre-treatments on the waste sludge resulted in supplementary 53 and 25% enhancement of biomethane, respectively. However, some research reported that excessive microwave pretreatment at high temperature may have adverse effect on methane yield due to the side effect of heat-induced inhibitors, such as phenolic compounds and furfural (Li et al., 2012). Steam explosion has been used to treat various kinds of lignocellulosic biomass for enhancement of methane production. After steam explosion, hemicellulose was hydrolyzed and lignin was reduced to a certain degree, thus resulting in degradation of lignin-carbohydrate complexes (Zhou et al., 2016). Moreover, steam explosion is often facilitated by additional acids, such as 6% SO_2_ (Vivekanand et al., 2014), diluted H_2_SO_4_ (Huang et al., 2015), and other chemicals. Liquid hot water pretreatment can enlarge the accessible surface area of substrate for higher cellulose degradability to cellulase. Under high temperature and pressure, water can penetrate into the interior of cell wall structure, solubilize hemicellulose, slightly remove lignin and hydrate cellulose. This method causes less corrosion to reactors and produces little amounts of byproducts and inhibitors, thereby has considerable potential of pentose recovery (Monlau et al., 2012; Yu et al., 2013).

### Chemical Pretreatment of Lignocellulose

Chemical pretreatment refers to the use of chemicals (e.g., acids, bases, oxidizing agents, organic solvents) to change physical and chemical characteristics of native lignocellulose. It has attracted the most research interest because of its higher efficiency on decreasing the resistant characteristics for better bioconversion performance. The positive effects of conventional chemical pretreatments are summarized in Table 4 and discussed below.

Acid pretreatment can prominently hydrolyze hemicellulose to mono saccharides, which will increase the pore size or volume of cell wall and make cellulose more susceptible to enzymatic degradation (Zhou et al., 2014). It can also disrupt lignin to a high degree, but only can dissolve little lignin in most cases. Considering the cost, toxicity by-products and equipment requirements, dilute acid is usually used for pretreatment in practical applications (Mussoline et al., 2013). Alkali is another popular pretreatment method. The function of alkali is believed to be two important effects: saphonication and solvation of lignin-carbohydrate linkages, which result in the enlargement and decrystallization of substrates (Van der Pol et al., 2014). The solvation can significantly remove lignin, acetyl groups and uronic acid of hemicellulose, which disrupts the lignin structure and breaks down the intramolecular bonds between lignin and other components. Therefore, the effectiveness of alkali pretreatment is associated closely with lignin content of lignocellulosic feedstock. Compared with traditional chemicals, ionic liquids possess some advantages of low toxicity, thermal stability, low hydrophobicity, enhanced electrochemical stability and so on. It has been proven to be positive on the improvement of biofuel production (Cao et al., 2017). During the pretreatment, ionic liquid can dissolve large amount of cellulose at mild conditions, and it is feasible to recover almost 100% of used liquid with high purity and leave little residues for the downstream anaerobic fermentation. The dissolution mechanism of cellulose in ionic liquids is the chemical interaction between its molecules and the oxygen and hydrogen atoms of cellulose hydroxyl groups (Xu et al., 2016). In the interaction, separation of oxygen and hydrogen atoms results in the opening of the hydrogen bonds between cellulose chains, which leads to the dissolution of cellulose (Feng and Chen, 2008). Then, dissolved cellulose can be regenerated by adding some specific chemical solvents which can precipitate cellulose from ionic liquid, such as ethanol, methanol, acetone, or water. The p mrecipitates have a higher enzymatic digestibility than native cellulose due to the changes in macro- and micro-structures. Crystallinity analysis of dissolved lignocellulose showed that the cellulose precipitates are different with either amorphous cellulose or cellulose II (Wahlstrom and Suurnakki, 2015).

### Biological Pretreatment of Lignocellulose

Biological pretreatment can be classified into three categories including fungal, microbial consortium and enzymatic pretreatment (Wei, 2016). Fungus has two specific systems including oxidative lignolytic system which exclusively attacks the phenyl bonds in lignin, and the hydrolytic enzyme system which degrades cellulose and hemicellulose. This pretreatment specifically degrades lignin, resulting in enhanced digestibility of cellulose (Kudanga and Roes-Hill, 2014). Cellulose is more recalcitrant to fungal attack than other components. Degradation of lignin and hemicellulose result in increased digestibility of cellulose, which is preferred for the following anaerobic fermentation. Microbial consortium pretreatment is conducted by microbes screened from natural environments in which rotten lignocellulosic biomass is the substrate. It is a complex microbial agent containing yeast and cellulolytic bacteria, heat-treated sludge, clostridium thermocellum, and mixture of fungi and composting microbes (Zhang et al., 2011). In contrast to fungal pretreatment, microbial consortium usually has high cellulose- and hemicellulose-degradation ability which mainly degrade cellulose and hemicellulose. Compared with physical and chemical pretreatment, the above two biological methods usually conducted in mild conditions which required far lower energy and chemicals input, and generated scarcely any inhibitors. However, the long pretreatment time limited the use of these processes in commercial applications. In addition, another important issue should be considered is that certain levels of carbohydrates are required by microbes during biological pretreatment, which resulted the competition between pretreatment and downstream biomethane production. Therefore, one major objective of biological pretreatment is to minimize the loss of carbohydrates and maximize the lignin removal. Enzymatic pretreatment prior to or in anaerobic digestion usually employs pure enzymes to accelerate the degradation of lignocellulose. The most commonly used enzymes mainly included cellulase, hemicellulose, and lignin-degrading enzyme, such as laccases and manganese peroxidase (Wei, 2016). These enzymes will help to release fermentable sugars from cellulose and hemicellulose to promote biomethane production from lignocellulosic biomass. However, the effect of enzymes in enhancing biogas production was minimal in most cases, and the cost of enzymes was high (Romano et al., 2011). Therefore, the application of enzymatic pretreatment has been limited.

Pretreatments have been proven to decrease the recalcitrance of native lignocellulose to obtain higher biomethane production. Generally, every pretreatment method has its major effect on different chemical or physical characteristics of the recalcitrance. Such as increasing accessible surface area, reducing crystallinity and polymerization, removing lignin content and so on (Table 4). However, all of these have the positive effect on the accessible area of lignocellulose. Therefore, grinding or milling is the most common first step for all pretreatment in biomethane production. Due to the complexity of lignocellulose chemical structures and different fermentation processes, the selection of pretreatment technologies must consider several factors, e.g., the type of lignocellulosic biomass, void the formation of by-products that are inhibitory to microorganisms, the downstream biological conversion processes, and the cost of pretreatment.

### Cell Wall Modification and Genetically Engineered Plants

Besides previous studies on pretreatment methods and management of anaerobic fermentation, some researches focusing on plant cell wall modification and even energy crops. Recently, performance of energy crops under various management practices has been extensively discussed elsewhere (Knoll et al., 2015; Cole et al., 2017). There are some strategies can be applied to modify plant cell wall *in vivo*, such as modification or interference of key enzymes in the biosynthesis pathways, expression of heterologous proteins or enzymes. These plant cell wall artificial modification would change the native biomass characteristics and got desired substrate (Vermerris and Abril, 2015). Li et al. (2018) over-expressed *Trichoderma reesei* β-1,4-D-glucosidasein the cell walls, and this significantly increased biomass porosity and reduced cellulose features (crystallinity or polymerization degree), resulting in enhanced biomass enzymatic hydrolysis. Fan et al. (2017) demonstrated that AtCesA8 -driven OsSUS3 expression in transgenic rice could reduce cellulose crystallinity and increase cell wall thickness, resulting in improved biomass saccharification. Another report found that lasalocid sodium pretreatment on *Arabidopsis* could upregulate type III peroxidase genes and change the cellular arrangement of hypocotyls, resulting in enhanced enzymatic saccharification (Okubo-kurihara et al., 2016). Moreover, research about synthesis and assembly mechanism of cellulose and hemicellulose provide great possibility to control and alter these processes in ways that would render the cell walls more easily. It can help to get better hydrolysis efficiency and considerable reduction of costly enzymes.

Compared to pretreatment, cell wall artificial modification or genetic engineered plants has more advantages because it does not require additional energy or chemicals input, produces fewer toxic by-products and causes less pollution to environment. At present, these researches have made some progress in improvement of bioethanol production. It is capable to create the desired breakthrough to overcome biomass recalcitrance. However, there are very limited studies on the changes in quality or composition of plant cell wall of tropical energy crops. An in-depth understanding of the precise plant cell wall structure and identification of the key affecting factors are still needed for optimizing the conversion of lignocellulosic biomass to biomethane in the future.

### Process Controlling and Optimization of Anaerobic Fermentation Process

Compared to mono-digestion, co-digestion of lignocellulose with animal feces shows significant potential for commercial biomethane production (Giuliano et al., 2013; Wei et al., 2014). Wang et al. (2017) reported 256.57 mL/g volatile solids methane was produced through the co-digestion of corn stalk and pig manure, which was increased by 17.4% than that using corn stalk mono-digestion. The higher efficiency of co-digestion mainly associated with process stability, e.g., optimal C/N ratio, ammonia reduction, and essential trace elements, which help maintain a steady condition for better performance of microorganisms to break down lignocellulose recalcitrance (Siddique and Wahid, 2018). Moreover, microbial reinforcement is another promising option to enhance enzymatic hydrolysis of lignocellulose and improve the biogas yield. Zhang et al. (2015) utilized 10% inoculation of *Acetobacteroides hydrogenigenes* as reinforcement, and got 19–23% increase of methane yield finally. Due to abundant enzymes (e.g., cellulase and xylanase) and sufficient nutrient content, digested manures have better adaptability in digesting lignocellulose for higher biomethane production (Gu et al., 2014). There are some basic requirements for anaerobic microorganism those degrade the particular lignocellulose in terms of environmental conditions and feed compositions inside the reactor (Mao et al., 2015). Different from pretreatment and cell wall modification, process optimization is an indirect strategy, which aims to provide a more reasonable environment for anaerobic bacteria to grow better and secrete more relevant enzymes to degrade lignocellulose more efficiently. For example, thermophilic microaerobic pretreatment (oxygen loads of 5 ml/g volatile solids substrate) on corn straw could promote the growth of aerobic microorganisms which secreted more hydrolytic enzymes in the early stage of the fermentation process. These enzymes would decrease cellulose crystallinity and cause substantial structural disruption of plant cell wall, which finally resulted in 16.24% higher methane production than that of untreated (Fu et al., 2015).

## Conclusions and Future Perspectives

Lignocellulose substrate shows great potential for biomethane production. Due to the inherent complicated recalcitrance of the plant cell wall, lignocellulosic biomass cannot be efficiently utilized during anaerobic digestion process. Generally, the direct factor affecting hydrolysis of biomass is the accessible surface area which is constructed by its chemical compositions that build a spatial network as a protection barrier. It plays more important role in reducing the initial enzymatic hydrolysis by limiting the substrate accessibility to enzymes or chemical regents. During the anaerobic digestion progress, accessible surface area will increase along with the removal of partial cell wall components, resulting in higher surface availability. Then the indirect factors such as chemical compositions (lignin, hemicelluloses, and acetyl group), and cellulose structure-relevant factors (cellulose crystallinity and polymerization degree), will play more important role in restricting the decomposition of substrate. As cross-linked polysaccharide networks, different influencing factors are not isolated, but closely related to each other and have synergetic impact on bioconversion. Although much is known about the structure of the plant cell wall and recalcitrance, there are still some fundamental questions that need further investigation, especially for the decomposition process in anaerobic digestion.

In the view of current knowledge, current strategies have positive contribution to improve biomethane production from lignocellulose. Pretreatment is still the most effective way to overcome the biomass recalcitrance, and selection of proper pretreatment method is very crucial for commercial biomethane production. However, high energy input and cost requirements of decomposing biomass recalcitrance are the main limitations. Cost-effective production of biomethane from lignocellulosic feedstocks depends much on significant improvement in both biomass quality and conversion efficiency. So, further studies should focus much more on investigating the relationship between the precise structure of cell wall recalcitrance and the key factor affecting the anaerobic digestion progress, which will be used to explore new methods to improve the biomethane production. Recently, some researches indicated that plant cell wall modification and artificial energy croup are fascinating strategies, which can improve quality, quantity and digestibility of traditional biomass material. With the development of biotechnology, transgenic plant may will be frequently applied in the anaerobic digestion system for biomethane production.

## Author Contributions

NX, SL, and JZ wrote the paper. FX and HJ provided the literature and data. JX, MJ, and WD contributed to the writing of the paper and the overall paper design.

### Conflict of Interest Statement

The authors declare that the research was conducted in the absence of any commercial or financial relationships that could be construed as a potential conflict of interest.
